# The interplay between work overload, trait motivation, and emotional exhaustion on job satisfaction and happiness

**DOI:** 10.59400/apr3071

**Published:** 2026-02-26

**Authors:** Fiona E. Raines, Shahnaz Aziz, Julia King, Kristin S. Allen

**Affiliations:** 1 Department of Psychology, East Carolina University, Greenville, NC 27858, USA; 2 SHL, Atlanta, Georgia, 30361 USA

**Keywords:** trait motivation, emotional exhaustion, work overload, happiness, job satisfaction

## Abstract

Job satisfaction and happiness have received considerable attention in recent literature, as the work landscape increasingly prioritizes and seeks to maximize employee well-being. Despite considerable extant research looking at organizational factors, individual antecedents of these desirable outcomes, such as motivation and burnout, have yet to be examined. In the present study, we seek to address this gap by applying the frameworks of the Job Demands-Resources model and Conservation of Resources theory to examine achievement motivation and emotional exhaustion (a key aspect of burnout) as predictors of job satisfaction and global employee happiness. A cross-sectional, secondary dataset sampling 844 working professionals via an Amazon Mechanical Turk survey was employed to investigate the interplay between environmental factors (i.e., work overload), individual factors (i.e., trait motivation and emotional exhaustion as a core component of burnout), and the outcomes of job satisfaction and happiness. Emotional exhaustion and anxiety motivation were negatively related to job satisfaction and happiness, while achievement motivation was positively related to them. Additionally, findings demonstrated evidence that work overload relates to job satisfaction and happiness through an emotional exhaustion statistical mediation pathway. Future researchers should confirm and expand on our findings by evaluating these relationships in longitudinal studies and more heterogeneous samples to examine temporal effects.

## 1. Introduction

Individual differences, such as trait motivation, play a critical role in the stress experience process by influencing whether a person appraises an external event as a stressor (Ganster and Perrewe, 2011). Most relevantly, achievement motivation refers to motivation that stems from the standards of excellence that are applied to one’s performance (Wigfield et al., 2021). For example, some individuals, such as those who lean toward anxiety motivation, may view a large project as a stressful demand, while those who veer toward achievement motivation might perceive the same project as an exciting new challenge. Similarly, employees experiencing symptoms of emotional exhaustion, a core component of burnout, may hold comparatively less bandwidth to take on new or complex tasks, predisposing them to appraise environmental stimuli as stressors. These relationships have been examined in extant literature via isolated personality traits, as they relate to burnout (e.g., Algelini, 2023; Alarcon et al., 2009; Swider and Zimmerman, 2010), and individual difference variables, as they relate to environmental appraisal (Alarcon et al., 2009; Tomaka and Magoc, 2021). In the present study, we seek to expand the field’s current understanding of this interplay by examining how two relevant individual difference variables, namely, emotional exhaustion and trait motivation, relate to job satisfaction and happiness at work. In doing so, we respond to a call to action by Demerouti et al. (2021) for additional high-quality research related to burnout, focusing on its foundational facet of emotional exhaustion.

### 1.1. Burnout and emotional exhaustion

Burnout is a psychological syndrome wherein perpetual exposure to stressful stimuli at work results in the depletion of intrinsic resources and related symptomatology (Edú-Valsania et al., 2022; Maslach et al., 2001; Shirom, 2011). This affective response can be observed through emotional exhaustion, or an experienced reduction in available physiological and psychological resources, which some researchers have suggested precedes other facets of burnout in most cases (Taris et al., 2005). Burnout has received considerable attention in the literature in recent decades (Demerouti et al., 2021), especially since the Covid-19 pandemic exacerbated emotional exhaustion symptoms, even for those in professions with already-problematic levels (Sexton et al., 2022).

Burnout has demonstrated harmful effects for both the individual and the organization, with widespread impact; thus, it has been a popular focus of literature in recent decades (Demerouti et al., 2021; Hakanen and Bakker, 2017; Shirom, 2011; Swider and Zimmerman, 2010). At the individual level, burnout has been linked to individual physical health outcomes, including higher prevalence rates of type II diabetes and increased risk of cardiovascular disease (Armon et al., 2010; Melamed et al., 2006; Shirom, 2011), compared to samples unafflicted with burnout. It has also been associated with musculoskeletal disorders, hospitalizations, depression, and anxiety (Armon et al., 2010; Melamed et al., 2006; Shirom, 2011; Bakker et al., 2014). However, little existing work has sought to understand how burnout affects individuals’ experiences of the workplace itself (namely, job satisfaction and happiness at work).

Emotional exhaustion, as one of three foundational elements of burnout (Maslach and Jackson, 1981), has been used in previous literature as a proxy measure for global burnout with sufficient validity and internal consistency (Cordes et al., 1997; Lee and Ashforth, 1996; Taris et al., 2005). Emotional exhaustion describes feelings of energy depletion or exhaustion (Maslach and Leiter, 2021). Emotional exhaustion itself has been empirically related to a decrease in growth intentions (Neneh, 2024), turnover intentions (Grobelna, 2021), and, specifically for teachers, student self-concept, interest, and achievement (Klusmann et al., 2022). It has also been linked to a decrease in motivation (Naidoo et al., 2012), along with work outcomes. For example, emotional exhaustion in teachers was associated with reduced emotional support, classroom organization, and student self-concept, interest, and achievement (Klusmann et al., 2022). Emotional exhaustion, as a depletion of employees’ psychological resources, has been traditionally conceptualized as an antecedent of harmful consequences (Lesener et al., 2020). However, here, we examine how it may act as a mediator in the work overload—job satisfaction and work overload—happiness relationships. By doing so, we seek to identify whether emotional exhaustion can explain how work overload predicts changes in job satisfaction and happiness, which past literature has found to be related to emotional exhaustion.

While the definition of burnout remains debated and thus ambiguous across literature and time, exhaustion is one ‘core constituent and necessary component’ of it which remains consistent (Demerouti et al., 2021; Schaufeli, 2021). Some scholars (i.e., Kristensen et al., 2005) even suggest that the three components of the definition employed in constructing the Maslach Burnout Inventory (MBI; Maslach and Jackson, 1981) include only one truly aligned construct—emotional exhaustion—while depersonalization describes a coping mechanism and reduced personal accomplishment is an outcome/effect. In alignment with this logic, we focus on emotional exhaustion in the present work as the independent third of the MBI, which is most likely to approach construct validity for burnout (Kristensen et al., 2005). Emotional exhaustion has also previously been demonstrated to act as a mediator in the emotional labor-physical and mental health relationships (Chen et al., 2022), making it of particular interest as we examine predictors of outcomes that do not overlap, but are likely related (namely, job satisfaction and happiness). Moreover, high levels of job demands are related more to exhaustion than cynicism or reduced professional efficacy (Mäkikangas et al., 2021).

### 1.2. Work overload

Burnout is frequently contextualized via the Job Demands-Resources model (JD-R), which has been honed and supported by substantive literature since it first appeared in 2001 (Bakker et al., 2014; Demerouti et al., 2001). The JD-R model posits that working conditions can be categorized into job demands and job resources; when demands outweigh resources, burnout occurs (Demerouti et al., 2001). Job demands are physical, social, or organizational components of a job that necessitate continued effort from the worker; they are more strongly related to exhaustion than engagement (Demerouti et al., 2001). This persistent effort, accordingly, leads to psychological detriments (Cole et al., 2012). Examples of job demands include role ambiguity, workload, and work-related pressure (Lee and Ashforth, 1996; Schaufeli and Buunk, 2002). Lee and Ashforth (1996) conducted a meta-analysis examining job demands in the context of human-services providers (e.g., teachers and counselors). They found role ambiguity, role conflict, role stress, stressful events, workload, and work pressure were the most predictive of burnout (Lee and Ashforth, 1996). In later years, Alarcon (2011) conducted research with employees from a wider variety of occupations and confirmed that role conflict workload and role ambiguity were some of the most influential antecedents of burnout. However, these relationships have not been applied to the outcome of happiness, which includes more than just job satisfaction. In the present study, we utilize the term ‘work overload’ to represent high job demands in accordance with the guidelines of Lee and Ashforth (1996). Thus, work overload will be applied as a predictor for both job satisfaction and happiness. Additionally, while past literature has primarily examined burnout as a broad outcome, we focus on emotional exhaustion as the most consistent facet of burnout across conceptualizations.

Conversely, job resources—which are related more strongly to engagement than exhaustion—are ‘those physical, psychological, social, or organizational aspects of the job’ that support the achievement of work goals, reduce job demands, or stimulate personal development (Demerouti et al., 2001). More recently, the JD-R model has been adapted to recognize the importance of individual factors, with some factors being even stronger predictors than social or contextual ones (Mazzetti et al., 2021). Following, we examine trait motivation as a potential individual factor that acts in this way as a resource when contextualized in the JD-R model.

### 1.3. Trait motivation

Motivational traits are “stable, cross-situational individual differences in preferences related to approach and avoidance, in the investment of goal-directed effort” that help to create opportunities in which skills are fostered (Heggestad and Kanfer, 2000, p. 3). Trait motivation, then, describes an individual’s propensity to be motivated by one of two distinct superordinate motivational trait complexes: achievement motivation and anxiety motivation (Kanfer and Heggestad, 1997). The achievement motivation complex is defined by approach orientation, personal mastery, and competitive excellence, such that an individual with this type of trait motivation is likely to willingly accept new challenges with an intention to learn and succeed (Chen et al., 2004). Extant literature has linked achievement motivation to traits such as conscientiousness, need for achievement, openness to experience, and learning-goal orientation (Chen et al., 2004). Further, achievement motivation is linked to mental health and academic achievement (Mahdavi et al., 2023). Alternatively, the anxiety motivation complex is characterized by avoidance motivation; individuals with this complex tend to experience emotional instability and negative affectivity, especially when faced with aversive stimuli (Chen et al., 2004; Kanfer and Ackerman, 2000).

The foundational constructs of this approach (i.e., achievement and anxiety motivation) map onto the behavioral activation system (BAS) and the behavioral inhibition system (BIS; Diefendorff and Mehta, 2007). Given its tendency to remain stable across situations, as well as its associations with the BAS and BIS, trait motivation has been identified as a construct that may provide a deeper understanding of individual differences than even the widely accepted five-factor model of personality (Diefendorff and Mehta, 2007). In the present study, assessments of both achievement motivation and anxiety motivation, as subscales of a trait motivation measure, are included to examine how individual differences might predict such individually and organizationally important outcomes as job satisfaction and happiness.

### 1.4. Job satisfaction

Job satisfaction is defined as “a pleasurable or positive emotional state resulting from the appraisal of one’s job or job experiences” (Locke, 1976, p. 1304). Previous literature has demonstrated a positive correlation of job satisfaction with both happiness and overall subjective well-being, making it especially relevant to occupational health psychology (Bowling et al., 2010). Additionally, it has shown a negative relationship with burnout, such that employees who experience burnout also have lower job satisfaction on average (Alarcon, 2011). Causal evidence from longitudinal research suggests burnout is the antecedent (Wolpin et al., 1991). For these reasons, job satisfaction is an essential facet of, and must be examined to support, employee well-being.

Job satisfaction contributes to health and well-being not only at the individual level, but also at the organizational level. Extant literature has found that job satisfaction is directly related to such organizational critical outcomes as job performance (Katebi et al., 2022), organizational citizenship behavior (Swaminathan and Jawahar, 2013), and turnover intention (Harrington et al., 2001), ultimately trickling down to save the organization time and personnel costs. Understanding the antecedents of and processes which contribute to job satisfaction is a valuable first step toward supporting the well-being of individuals and their organizations.

### 1.5. Happiness

Another contributor to overall well-being for both individuals and organizations that has not yet been widely studied as an outcome of emotional exhaustion or trait motivation is happiness. Scholars have yet to reach a consensus regarding the operational definition of happiness (Cummins, 2012), defined by Diener et al. (1991, p. 123) as “relatively frequent positive affect and infrequent negative affect.” However, ‘happiness’ is consistently used as an umbrella term to describe positive feelings about the self, generally as a word synonymous with overall subjective well-being, or as an average of positive and negative affect (Cummins, 2012). Happiness is included in the present study to represent a measure of subjective well-being which is not specific to the work context, but rather includes influence from work and other facets of life; we refer to it here as ‘global’ to illustrate that it encompasses multiple life domains. A meta-analysis by Lyubomirsky et al. (2005) found that regardless of definition or measure, happiness is connected to successful outcomes across all major life domains (i.e., work, love, health) and to desirable attributes and behaviors such as creativity, prosocial behavior, and coping; this makes happiness a covetable trait in almost all contexts. Empirical evidence suggests happiness is influenced by work and can also affect work success (Rodriguez-Munoz and Sanz-Vergel, 2013).

Previous research has suggested a negative link between burnout and happiness; however, the method used to measure happiness contained only a single item (Schaufeli et al., 2009). Accordingly, a more robust measure of happiness was employed in the present study to contribute to extant literature by building upon previous limitations to provide additional evidence for this conclusion. Additionally, we examine how the Conservation of Resources theory and Job Demands-Resources model can be extended to apply to happiness via the resources of achievement motivation and anxiety motivation.

## 2. Present study

Given the theoretical foundations established through the Conservation of Resources (COR) theory and Job Demands-Resources (JD-R) model (see below for details), in the present study, we seek to examine how individual differences, as personal resources, can impact job satisfaction and happiness. Following, we employ measures of emotional exhaustion and trait motivation (including sub-measures of both achievement and anxiety motivation) to examine how individual differences influence work-relevant outcome variables that affect both the individual and the organization. In doing so, we address a gap in the literature by 1) expanding the applications of the COR theory and the JD-R model to a happiness outcome; and 2) conceptualizing emotional exhaustion as a mediating factor. By reconceptualizing emotional exhaustion as a statistical pathway through which work overload impacts desirable outcomes, rather than as a direct antecedent (Naidoo et al., 2012) or an outcome (Lesener et al., 2020), we seek to extend the ways in which COR theory and JD-R model have been applied to outcomes as a function of resource availability. Additionally, by examining happiness as a more global indicator of well-being than job satisfaction, we seek to extend the conclusions of extant literature and examine whether emotional exhaustion, trait motivation, and work overload are related to experiences of life outside of work.

### 2.1. Conservation of resources theory

The Conservation of Resources theory (COR; Hobfoll, 1989) asserts that individuals whose resources are depleted or unavailable are more likely to experience increases in strain and negative affective outcomes. It follows logically that those experiencing emotional exhaustion, whose personal resources are weakened by recurrent exposure to work stress (Shirom, 2011), will appraise work experiences as more stressful. Previous literature has directly linked burnout to decreased job satisfaction (e.g., Baruch-Feldman et al., 2002; Demerouti et al., 2001), for which we seek to provide supporting evidence and calibrate our findings. Additionally, extant work has related burnout to decreased well-being (Schaufeli et al., 2009); however, no existing literature has examined the potential linkage between emotional exhaustion, as a core component of burnout, and a more global measure of happiness. Thus, we hypothesize that:

**H1.** *Emotional exhaustion will be negatively related to job satisfaction*.
**H2.** *Emotional exhaustion will be negatively related to happiness*.

Literature has also found significant correlations between achievement motivation and job satisfaction (*r* = 0.65 and *r* = 0.44), and between anxiety motivation and job satisfaction(*r* = −0.40 and *r* = −0.48; Ferris et al., 2013; Briki, 2018). These correlations support the conceptualization of achievement motivation as a resource and anxiety motivation as a threat to personal resources, thereby strengthening the assertion that individuals high in achievement motivation are more likely, and individuals high in anxiety motivation are less likely, to experience positive affect at work. To provide bolstering evidence that trait motivation is related to job satisfaction and expand this conceptualization to the happiness outcome, we hypothesize that:

**H3.** *Trait motivation will be significantly related to job satisfaction such that:*
**H3a.** *Achievement motivation will be positively related to job satisfaction*.
**H3b.** *Anxiety motivation will be negatively related to job satisfaction*.
**H4.** *Trait motivation will be significantly related to happiness such that:*
**H4a.** *Achievement motivation will be positively related to happiness*.
**H4b.** *Anxiety motivation will be negatively related to happiness*.

### 2.2. Job demands-resources model

Another theoretical groundwork for these hypotheses lies within the Job Demands-Resources model (JD-R; Bakker et al., 2014) which, as mentioned, posits that resources can act as a buffer against the negative effects of high job demands (such as strain experiences and emotional exhaustion), and that personal traits/individual differences can qualify as resources when they lead to positive outcomes. The COR theory and the JD-R model both emphasize the importance of maintaining resources (such as achievement motivation) to promote well-being. A healthy balance between job demands and resources can maximize employee well-being and performance, while an excess of job demands which are not offset by resources can lead to emotional exhaustion. Alternatively, the toll of high job demands (i.e., work overload) creates a deficit for employees which cannot always be recovered through the presence of resources, thereby resulting in burnout and, more specifically, emotional exhaustion (Bakker et al., 2014; Demerouti et al., 2001).

The JD-R model can provide additional contextualization and support for the aforementioned hypotheses. With emotional exhaustion being conceptualized as a drain on personal resources, the application of JD-R would lead to the conclusion that job satisfaction and happiness would be negatively impacted (H1 and H2). Similarly, achievement trait motivation is here established as a personal resource, leading to increases in job satisfaction and happiness (H3a and H4a), while anxiety trait motivation is conceptualized as a personal appraisal process that leads to higher demands that cannot be easily offset by personal resources (H3b and H4b). In each scenario, a change in the relationship between demands and resources as a function of individual differences leads to outcomes that affect both the individual and the organization, following the reasoning of the JD-R model.

Additionally, extant research employing the JD-R model has indicated that 1) work overload predicts burnout [anonymized for peer review]; 2) burnout and job satisfaction are causally related, with burnout causing changes in job satisfaction (Wolpin et al., 1991); and 3) burnout predicts unique and incremental variance across indicators of well-being, including life satisfaction and depressive symptoms (Hakanen and Schaufeli, 2012). We apply and synthesize these findings to hypothesize that emotional exhaustion, as a facet of burnout particularly relevant to the depletion of resources, will predict changes in job satisfaction and happiness (here used as a global indicator of well-being) due to high job demands (i.e., work overload):

**H5.** *Emotional exhaustion will mediate the relationship between work overload and job satisfaction*.
**H6.** *Emotional exhaustion will mediate the relationship between work overload and happiness*.

## 3. Materials and methods

### 3.1. Data collection

This study adhered to ethical guidelines and principles, including obtaining informed consent from participants, maintaining confidentiality and anonymity, and ensuring data integrity. Secondary data analysis was employed on a dataset collected in 2017 via Amazon Mechanical Turk (MTurk). The dataset included responses from 1054 employees across the United States. All participants necessarily had one job (i.e., primary employment) which was being supplemented by research participation work on MTurk. Data collection efforts were originally led by a global organization that specializes in providing best practice insights and technology to leaders across various business domains.

### 3.2. Participants

A total of 844 participants were included in the analysis, after 210 participants were removed due to incomplete surveys or incorrect answers to instructed response items. Respondents ranged from 18 to 73 years of age (M = 34.57), with the majority being male (57%; 43% female; >1% preferred not to answer). Regarding the highest level of education achieved, most respondents (48%) reported holding a bachelor’s degree; 19% reported having some college or university experience, but no degree, and 17% had a master’s or professional degree. Most respondents identified as Caucasian (75%), while the remaining respondents identified as Asian (9%), African American (6%), more than one race (6%), Hispanic (4%), or other (1%). Participant employment spanned various industries, including but not limited to education (14%), technology (14%), healthcare (11%), government or non-profit (10%), retail (8%), and manufacturing (7%) roles. Twenty-nine percent self-reported management-level positions, and 1% were in executive-level roles. The average role tenure across participants was 4.5 years. The average organizational tenure across participants was 5.8 years.

### 3.3. Measures

#### 3.3.1. Work overload scale

A measure developed for research purposes by a global leader in psychometric science was used to capture work overload. The measure was validated as part of a study intended to define a taxonomy of organizational context factors as they applied to leader performance (Johnson and Arad, 2018). It included items related to various dimensions within the broader context of the organization, team, and role (Johnson and Arad, 2018). The scale included three items, such as “I am not given enough time to do what is expected of me.” Respondents were asked to rate each item on a 7-point scale ranging from 1 (strongly disagree) to 7 (strongly agree). The work overload scale demonstrated acceptable internal consistency (α = 0.81).

#### 3.3.2. Trait motivation questionnaire

Kanfer and Ackerman’s (2000) Motivational Trait Questionnaire (MTQ) was employed to assess the motivational traits of achievement motivation and anxiety motivation. The achievement motivation subscale included 16 items, such as “I thirst for knowledge,” while the anxiety motivation subscale included 19 items, such as “I am able to remain calm and relaxed in stressful situations” (reverse-coded). Respondents were asked to rate each item on a 7-point scale ranging from 1 (very untrue of me) to 7 (very true of me). The MTQ demonstrated acceptable internal consistency in the present study (α = 0.94 for the achievement motivation subscale and α = 0.94 for the anxiety motivation subscale).

#### 3.3.3. Emotional exhaustion scale

A six-item version of the Maslach Burnout Inventory (MBI; Maslach and Jackson, 1981) was utilized to assess emotional exhaustion. The emotional exhaustion subscale is a sufficient proxy for global burnout (Cordes et al., 1997; Lee and Ashforth, 1996; Taris et al., 2005); thus, it was employed to reduce survey fatigue and to represent a core and consistently cited burnout component. A sample item was “I feel burned out from my work.” Participants rated each item on a 7-point frequency scale ranging from 1 (never) to 7 (every day). The emotional exhaustion scale showed acceptable internal consistency (α = 0.93).

#### 3.3.4. Job satisfaction survey

The Job Satisfaction Survey (JSS; Spector, 1985) was used to measure job satisfaction. The JSS originally included 36 items; to reduce potential survey fatigue, we omitted the contingent rewards, operating procedures, and communications subscales. Additionally, all subscales (except supervision) were reduced to 3 items for a total of 19 items (e.g., “I like doing the things I do at work”). The shortened JSS scale held acceptable internal consistency (α = 0.92).

#### 3.3.5. Happiness questionnaire

The unidimensional Oxford Happiness Questionnaire (OHQ) was employed to assess happiness (Hills and Argyle, 2002). This measure consisted of 29 items to be endorsed on a 7-point scale ranging from 1 (strongly disagree) to 7 (strongly agree). A sample item includes “I feel that life is very rewarding.” The OHQ showed acceptable internal consistency in the present study (α = 0.94).

### 3.4. Data analysis

Demographic variables were assessed and used to describe participants. Descriptive statistics (i.e., means and standard deviations), along with a correlation matrix, were generated to examine and compare all key study variables, including work overload, trait motivation, emotional exhaustion, job satisfaction, and happiness. The associated measures for each of these variables were examined for internal consistency using the psychometric acceptability standard for reliability (α = 0.70 or above; Nunnally and Bernstein, 1994).

Linear regression models were used to separately examine the relationships of all predictors with job satisfaction and happiness (H1, H2, H3a, H3b, H4a, H4b). Then, the mediating effect of emotional exhaustion on the relationship between work overload and job satisfaction was modeled and run (H5). Finally, the mediating effect of emotional exhaustion on the relationship between work overload and happiness was modeled and run (H6). Using Hayes’ PROCESS software, the total effect of work overload was decomposed into direct and indirect effects in each model (H5 and H6). Bootstrapped confidence intervals, which result in higher power than the normal theory approach (Hayes, 2013), were used to test the indirect effect of emotional exhaustion on both outcomes for significance. Given the simplicity of the mediation model, with a single mediational pathway, separate regression models were preferred over a structural path model for clarity in the interpretation of effects, while maintaining methodological rigor and incremental assessment of the hypotheses.

## 4. Results

### 4.1. Descriptive statistics and correlations

Descriptive statistics, Cronbach’s coefficient alphas, and zero-order correlations for all substantive and demographic variables are displayed in Table 1. Cronbach’s coefficient alphas ranged from 0.81 to 0.94, all exceeding the 0.70 accepted field threshold, thereby demonstrating an acceptable level of internal consistency (Nunnally and Bernstein, 1994). Work overload was not significantly correlated with the achievement motivation subscale (*r* = −0.02, *p* = 0.66), but it was significantly and positively correlated with the anxiety motivation subscale (*r* = 0.22, *p* < 0.001) and emotional exhaustion (*r* = 0.47, *p* < 0.001). Furthermore, the achievement subscale of trait motivation was significantly negatively related to emotional exhaustion (*r* = −0.14, *p* < 0.001), while the anxiety motivation subscale was significantly positively related to it (*r* = 0.39, *p* < 0.001).

In addition, work overload was significantly negatively related to happiness (*r* = −0.22, *p* < 0.001) and job satisfaction (*r* = −0.40, *p* < 0.001). The achievement motivation subscale was significantly positively related to happiness (*r* = 0.42, *p* < 0.001) and job satisfaction (*r* = 0.26, *p* < 0.001). The anxiety motivation subscale was significantly negatively related to happiness (*r* = −0.52, *p* < 0.001) and job satisfaction (*r* = −0.28, *p* < 0.001). Finally, emotional exhaustion was significantly negatively related to happiness (*r* = −0.53, *p* < 0.001) and job satisfaction (*r* = −0.58, *p* < 0.001). Intercorrelations amongst the trait motivation subscales were also assessed. Achievement motivation was significantly negatively related to anxiety motivation (*r* = −0.26, *p* < 0.001).

### 4.2. Hypothesis tests

H1 and H2 were supported by linear regression modeling, with emotional exhaustion negatively predicting both job satisfaction (*r* = −0.58, *p* < 0.001, 95% CI [−0.64, −0.53]) and happiness *(r* = −0.53, *p* < 0.001, 95% CI [−0.59, −0.48]).

Results also indicated support for H3a, H3b, H4a, and H4b, such that the complexes of trait motivation related to both job satisfaction and happiness in the predicted directions. Achievement motivation was positively related to job satisfaction (*r* = 0.26, *p* < 0.001, 95% CI [0.20, 0.33]), while anxiety motivation was negatively related to it (*r* = −0.28, *p* < 0.001, 95% CI [−0.34, −0.21]), providing support for H3a and H3b, respectively. Similarly, achievement motivation positively predicted happiness (*r* = 0.42, *p* < 0.001, 95% CI [0.36, 0.48]), while anxiety motivation negatively predicted it (*r* = −0.52, *p* < 0.001, 95% CI [−0.58, −0.47]).

Lastly, H5 and H6 were both supported, indicating that emotional exhaustion mediated the negative predictive relationship of work overload with both job satisfaction and happiness. The mediation models were tested using Hayes’ PROCESS software. Bootstrapping was implemented as a non-parametric resampling method to increase power above and beyond traditional theory approaches (Hayes, 2013).

**Figure 1.**
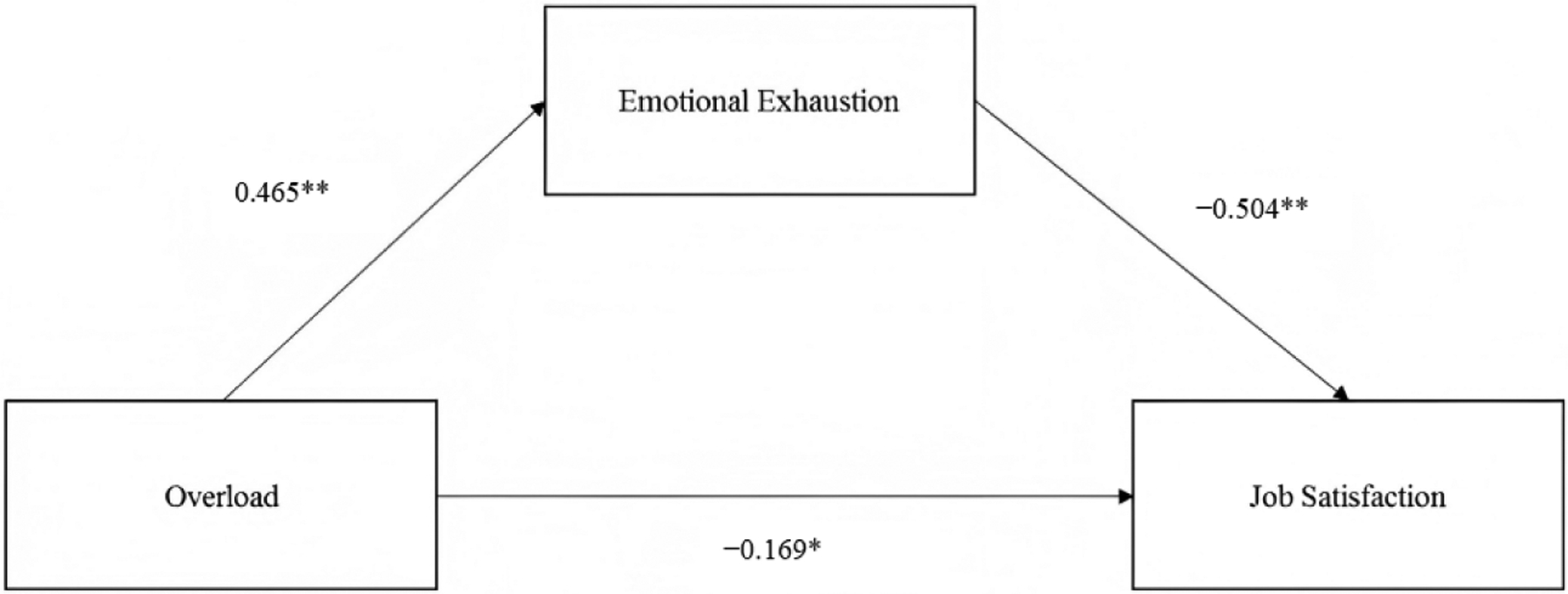
Mediation model predicting job satisfaction. Note: *p < 0.05, **p < 0.001.

Results yielded support for a mediated model to predict job satisfaction (Figure 1). The standardized total effect of work overload on job satisfaction (β = −0.403) was partitioned into a direct effect of −0.169, 95% CI [−0.230, −0.108] and an indirect effect of −0.235, 95% bootstrapped CI [−0.281, −0.191] (unstandardized coefficients are found in Table 2).

**Figure 2.**
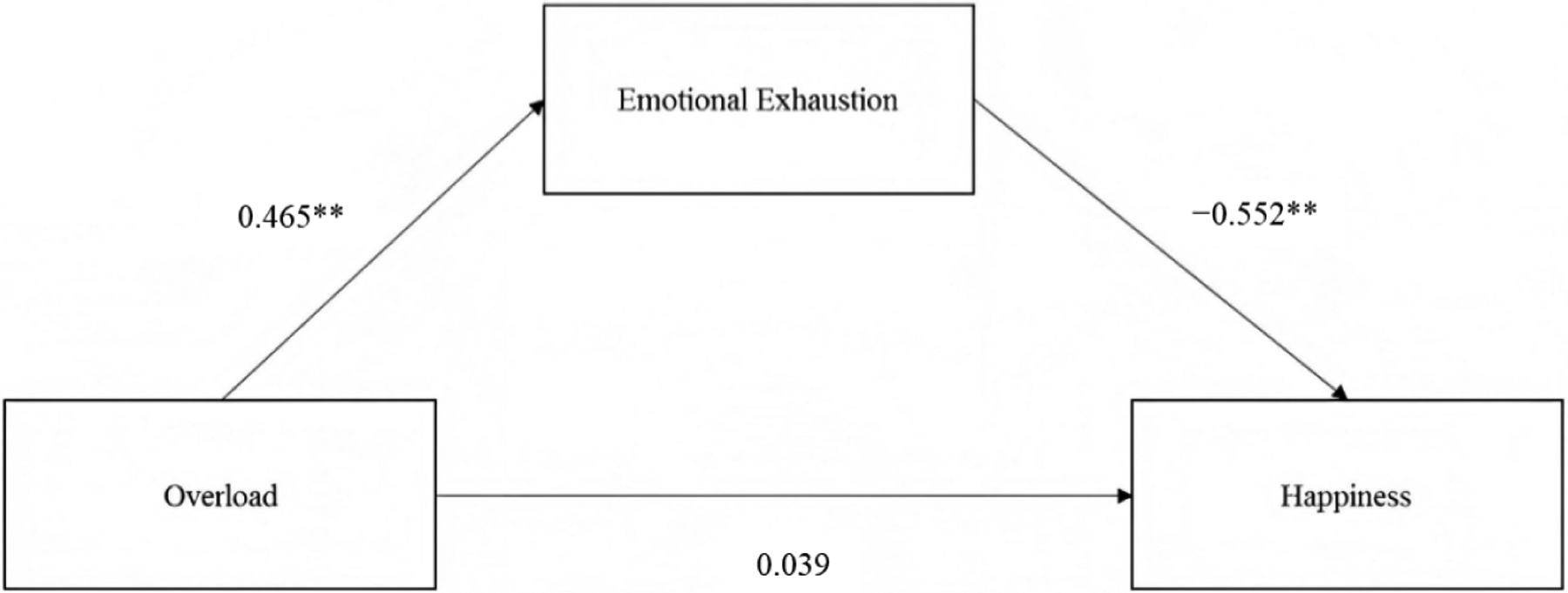
Mediation model predicting happiness. Note: **p < 0.001.

The results also yielded support for a mediated model in predicting happiness (Figure 2). The standardized total effect of work overload on happiness, β = −0.218, was partitioned into a direct effect of 0.039, 95% CI [−0.026, 0.103] and an indirect effect of −0.257, 95% bootstrapped CI [−0.307, −0.210] (unstandardized coefficients are found in Table 3). In this model (H6), the direct effect did not maintain significance upon accounting for the indirect effect of emotional exhaustion.

## 5. Discussion

In the present study, we explored the relationships between individual difference predictors with job satisfaction and happiness—two outcomes which have widespread and lasting effects at both the individual and organizational levels. As predicted, emotional exhaustion and anxiety motivation were significantly and negatively related to both job satisfaction and happiness, while achievement motivation was significantly and positively related to the same outcomes.

The process through which work overload is related to job satisfaction and happiness was also investigated. Based on the assertions of both the JD-R model and the COR theory, we hypothesized that work overload would not directly result in reduced job satisfaction or happiness, but would instead affect these variables through a related increase in emotional exhaustion. The mediation models represent associative pathways consistent with theory, not definitive causal mechanisms. Support for both statistical mediation models bolstered the notion that job demands affect individuals over time by depleting them of important resources, though the models should be further assessed with longitudinal data to examine causal mediation. Accordingly, it reinforced previous findings which demonstrated clear relationships between work overload, facets of burnout (such as emotional exhaustion), and job satisfaction (Wolpin et al., 1991; Hakanen and Schaufeli, 2012).

Moreover, we contributed to the current knowledge via two new findings: first, by expanding the application of the JD-R model and the COR theory to a global measure of happiness as a novel outcome of work overload and trait motivation; and second, by asserting that emotional exhaustion is a statistical pathway through which work overload relates to both job satisfaction and happiness, individually. Identifying emotional exhaustion as a mediator in these processes provides new insight that organizations may be able to mitigate the negative impacts of work overload by taking steps to prevent emotional exhaustion in employees before it affects job satisfaction, happiness, or other organizationally important outcomes. Examples of strategies that have demonstrated effectiveness to reduce emotional exhaustion without reducing workload include, for example, mindfulness-based stress reduction, cognitive behavioral strategies, meaning-centered therapy, and compassion training (McFarland and Hlubocky, 2021). By working to prevent and/or attenuate emotional exhaustion, organizational leadership can foster job satisfaction and happiness, thereby securing more positive outcomes for themselves and their employees in the future.

### 5.1. Theoretical contributions to applied psychology

The present findings advance theory by repositioning happiness within the Job Demands-Resources model and the Conservation of Resources theory. Whereas happiness has traditionally been interpreted as a trait-based resource or outside the scope of occupational stress models, we examine it as an outcome that is, at least in part, dependent upon work overload. By framing job satisfaction and broader happiness factors as potentially work context- and resource-dependent outcomes, we position each as an indicator of resource sufficiency at work and sustainable functioning both within (job satisfaction) and outside of (happiness) work, extending traditional applications of the JD-R and COR to work-specific contexts. Thus, we extend these theoretical frameworks to not only confirm how job demands and resources relate to emotional exhaustion, but also further their applications to investigate when and why resource sufficiency translates to desirable well-being outcomes. Similarly, we reconceptualize emotional exhaustion as a regulating factor that can facilitate or impede job demands’ impacts on job satisfaction and happiness, rather than as a terminal outcome. By doing so, we introduce a model of happiness which is partially contingent upon job-demands balance in the workplace rather than being wholly trait-dependent, and provide the groundwork for a potential mechanism through which the relationship can be shaped. Though the present findings align with traditional JD-R expectations, they refine the model by extending the outcome to broader measures of well-being and reinterpreting emotional exhaustion as a potential mechanism through which the work context predicts happiness. By doing so, they extend practical applications of work overload beyond organizational productivity to well-being on a more personal level.

### 5.2. Limitations and future directions

While our findings contribute to current literature by uncovering two significant construct relationships that have yet to be studied in the body of extant knowledge, additional work is needed to address limitations and confirm our results. For example, these findings may be limited in generalizability due to the sample being recruited and surveyed via Amazon Mechanical Turk. The utilization of online sampling raises doubts about the quality of the data. Resultantly, the sample was primarily Caucasian, male, and from the United States. Furthermore, concerns may arise about whether participants engaged attentively in the tasks provided from start to finish, without bot usage, random responses, or inattention. The findings presented here would thus benefit from confirmatory analysis with more diverse samples, including samples outside of the United States and samples that are not recruited and surveyed via an online platform. Relatedly, the present data was collected in 2017, which may raise concerns regarding its relevance. However, burnout and its facets have demonstrated relative stability (Mäkikangas et al., 2021), and emotional exhaustion has shown stability or increased severity over time (Dicke et al., 2022). Despite this theoretical stability, emotional exhaustion was studied more frequently after the Covid-19 pandemic (i.e., Hwang et al., 2021; Sexton et al., 2022; Bleck and Lipowsky, 2022; Barello et al., 2021), especially among healthcare and education professionals. By providing data that examines emotional exhaustion prior to the pandemic, we offer a baseline to which more recent, post-pandemic work can be compared.

Additionally, while previous research suggests cross-sectional experimentation is ideal for investigating boundary conditions (i.e., moderators) on established relationships (Spector, 2019), it is not appropriate for making causal inferences. Thus, as we cannot examine temporal factors, a causal inference cannot be drawn from the present study data. This limitation is especially applicable to H5 and H6, which use mediation analysis to test hypotheses that suggest how an antecedent variable transmits its effect on a consequent variable (Hayes, 2015). For our mediation analyses and their related conclusions to be correctly interpreted, confirmation that work overload is in fact an antecedent to changes in job satisfaction or happiness (rather than an outcome) is necessary. However, some researchers suggest it is still acceptable to test mediation models despite such limitations (Hayes, 2013; Spector, 2019) and recognize that even longitudinal research can lead to erroneous conclusions when the time lag is not appropriately specified for the phenomenon under study (Spector, 2019). Relatedly, some findings indicate that the emotional exhaustion component of burnout precedes the others (cynicism and reduced personal accomplishment; Taris et al., 2005), making its measure an appropriate proxy for burnout itself (Cordes et al., 1997; Lee and Ashforth, 1996; Taris et al., 2005). However, additional research is crucial to 1) better understand temporal effects on burnout as a whole and the associated causal sequence; and 2) examine the relationships of individual difference variables with job satisfaction and happiness outcomes using full (rather than abbreviated) or otherwise compacted measures of burnout. In doing so, future researchers can elucidate the nuances and causal directions of the significant relationships found in the present study.

Future studies may also include job performance as an additional outcome variable. Some research suggests a direct linkage between emotional exhaustion as a component of burnout and job performance, though the relationship is weak (Hakanen and Bakker, 2017; Taris, 2006). However, job satisfaction has also been directly related to job performance (Judge et al., 2001), as well as emotional exhaustion (and trait motivation) with job satisfaction, as demonstrated in the present study. Thus, researchers may seek to further our understanding by examining whether facets of burnout could instead act as pathways through which work overload affects job performance using mediation models.

### 5.3. Practical implications

The present study findings indicated that emotional exhaustion, a component of burnout, was significantly and negatively related to both job satisfaction and happiness in a diverse sample of workers across industries. Additionally, work overload’s harmful effects on both job satisfaction and happiness were mediated by an increase in emotional exhaustion. Based on these findings, employees who are experiencing the beginnings of burnout as a whole are also more likely to feel less satisfied with their jobs and less happy in general.

Previous work has negatively linked job satisfaction with turnover, indicating that employees with lower job satisfaction are more likely to leave their positions and organizations (Griffeth et al., 2000). Doing so costs organizations additional time and personnel costs that could be avoided. Decreases in job satisfaction have also shown predictive value for job performance (Katebi et al., 2022) and organizational citizenship behavior (Swaminathan and Jawahar, 2013). Relatedly, happiness has been associated with improved work success, income, decision-making (at the managerial level), and job performance (Fisher, 2010). Reflection upon these outcomes illuminates the unquestionable importance of bolstering job satisfaction and happiness, for the benefit of both individual and organizational health.

The study’s novel findings—especially that emotional exhaustion statistically mediates the relationships of work overload with these outcomes—give insights into how organizations can proactively work to manage employee job satisfaction and happiness levels. Taking this knowledge into account, organizations may choose to incorporate screenings for emotional exhaustion symptomatology into their annual procedures or provide training opportunities for leadership that address the identification and alleviation of emotional exhaustion (e.g., job crafting). Furthermore, organizational leadership should consider implementing proactive intervention strategies, where appropriate, to increase resilience (e.g., through PsyCap) or reduce stress (e.g., via mindfulness) to mitigate the effects of developing emotional exhaustion and help employees manage stressful workloads (Luthans et al, 2006; Roeser et al., 2013). Combined individual- and organizational-level interventions aimed at preserving employee resources, avoiding emotional exhaustion, and preventing the worsening of emotional exhaustion symptoms demonstrate the highest effectiveness, especially through reduced workload. Additional research is needed to identify which interventions have the best outcomes (Bes et al., 2023). However, research has identified some interventions that are effective within specific fields. For example, among mental health workers, the adoption of three key organizational priorities can be effective in reducing burnout as a whole. These include having a culture of person-centered care over productivity and metrics, engaging in practices to overcome bureaucracy, and providing opportunities for professional development and self-care (Rollins et al., 2021). Based on the present findings, we recommend that interventions focus on 1) reducing chronic work overload for employees and 2) preventing and mitigating emotional exhaustion in order to maximize job satisfaction and happiness. Evidence has shown that focusing on reasonable levels of job demands in job design, especially in times of crisis (i.e., the Covid-19 pandemic) during which employees experienced heightened demands in all facets of life, is recommended (Demerouti and Bakker, 2022). Leaders, who directly influence their employees’ demands and resources, and the impact of those demands and resources on well-being (Tummers and Bakker, 2021), should also be trained to notice signs of emotional exhaustion.

## 6. Conclusion

By examining the relationships of emotional exhaustion, trait motivation, and work overload with job satisfaction and global employee happiness, in the present study, we have provided confirmatory evidence for several assertions in extant literature and contributed new knowledge to the field. We found that work overload relates directly to emotional exhaustion, and that employees experiencing this facet of burnout tend to experience less job satisfaction. Additionally, we expanded the applications of the JD-R model and the COR theory to include happiness as a positive outcome of sufficient resource supply at work. In the process, we also identified emotional exhaustion as a mediator in the linkage between work overload and happiness, providing a foundation for practitioners to develop actionable pathways through which organizations can mitigate the harmful effects of work overload on their employees before its effects spiral into substantial organizational costs. By better understanding emotional exhaustion and striving to prevent it, organizational leaders may be able to enhance job satisfaction and overall happiness.

## Figures and Tables

**Table 1.** Descriptive statistics and intercorrelations.

Variable	1	2	3	4	5	6	7	8	9
1. EE	(0.93)								
2. ACM	−0.14**	(0.94)							
3. AM	0.39**	−0.26**	(0.94)						
4. OV	0.47**	−0.02	0.22**	(0.81)					
5. HA	−0.53**	0.42**	−0.52**	−0.22**	(0.94)				
6. JS	−0.58**	0.26**	−0.28**	−0.40**	0.56**	(0.92)			
7. Age	−0.03	0.05	−0.15**	0.03	−0.01	0.05	-		
8. Tenure (O)	0.00	0.04	−0.11**	0.01	0.01	0.06	0.49**	-	
9. Tenure (R)	0.03	0.00	−0.07	0.01	−0.01	0.00	0.48**	0.68**	-
Mean	20.80	86.36	73.35	10.48	136.8	88.95	34.57	69.56	54.54
*SD*	9.39	14.67	22.59	4.50	27.33	20.70	9.62	67.25	57.13

Note: *N* = 844. Entries on the main diagonal are Cronbach’s alphas. EE = Emotional Exhaustion subscale; ACM = Motivation Related to Achievement subscale; AM = Motivation Related to Anxiety subscale; OV = Overload; HA = Happiness; JS = Job Satisfaction; Tenure (O) = Organizational Tenure (Months); Tenure (R) = Role Tenure (Months).

** *p* < 0.001.

**Table 2.** Unstandardized model coefficients for the mediation model predicting job satisfaction.

Consequent								
*M* (Emotional exhaustion)					*Y* (Job satisfaction)			
Antecedent		Coeff.	*SE*	*p*		Coeff.	*SE*	*p*
X (OV)	*a*	0.971	0.064	<0.001	*c*’	−0.776	0.143	<0.001
*M* (EE)		-	-	-	*b*	−1.111	0.069	<0.001
Constant	*i*1	10.615	0.726	<0.001	*i*2	120.198	1.620	<0.001
		R^2^ = 0.217				R^2^ = 0.362		
		*F*(1,842) = 232.739, *p* < 0.001				*F*(2,841) = 238.117, *p* < 0.001		

Note: *N* = 844. OV = Overload; EE = Emotional Exhaustion subscale.

**Table 3.** Unstandardized model coefficients for the mediation model predicting happiness.

Consequent								
*M* (Emotional exhaustion)					*Y* (Happiness)			
Antecedent		Coeff.	*SE*	*p*		Coeff.	*SE*	*p*
X (OV)	*a*	0.971	0.064	<0.001	*c*’	0.235	0.200	0.241
*M* (EE)		-	-	-	*b*	−1.606	0.096	<0.001
Constant	*i*1	10.615	0.726	<0.001	*i*2	167.697	2.261	<0.001
		R^2^ = 0.217				R^2^ = 0.286		
		F(1,842) = 232.739, *p* < 0.001				F(2,841) = 168.432, *p* < 0.001		

Note: *N* = 844. OV = Overload; EE = Emotional Exhaustion subscale.

## Data Availability

Research data is available but not shared because we did not obtain permission from survey participants to share the data. In our Data Protection Notice (refer to Institutional Review Board Statement), it says that we will anonymize and store the data.
